# Circulating miRNAs as diagnostic biomarkers for multiple myeloma and monoclonal gammopathy of undetermined significance

**DOI:** 10.1002/jcla.23233

**Published:** 2020-02-10

**Authors:** Jia Li, Man Zhang, Chengbin Wang

**Affiliations:** ^1^ Medical School of Chinese PLA & Medical Laboratory Center The First Medical Center of Chinese PLA General Hospital Beijing China; ^2^ Clinical Laboratory Medicine Beijing Shijitan Hospital Capital Medical University Beijing China; ^3^ Beijing Key Laboratory of Urinary Cellular Molecular Diagnostics Beijing China

**Keywords:** biomarker, microRNA, molecular diagnosis, monoclonal gammopathy of undetermined significance, myeloma

## Abstract

**Background:**

Multiple myeloma (MM) is still an incurable hematological malignancy evolved from asymptomatic monoclonal gammopathy of undetermined significance (MGUS). New evidence suggests that circulating microRNAs (miRNAs) can serve as stable diagnostic biomarkers for MM and MUGS.

**Methods:**

Serum miRNAs in MM patients, MUGS patients, and healthy controls (HC) were performed by Agilent Bioanalyzer 2100. MicroRNAs in MM detected as promising biomarkers were validated by using quantitative real‐time PCR (qRT‐PCR). Receiver operator characteristic (ROC) curve and multivariate logistic analysis were used to evaluate the diagnostic value of miRNAs for MM and MUGS.

**Results:**

In microarray analysis, the top ten differential expressed miRNAs in MM included miR‐134‐5p, miR‐107, miR‐15a‐5p, miR‐5159‐3p, miR‐1914‐3p, miR‐4723‐3p, miR‐5588‐3p, miR‐6893‐3p, miR‐7106‐3p, and miR‐6722‐5p. Three up‐regulated miRNAs (miR‐134‐5p, miR‐107, and miR‐15a‐5p) were further validated. The elevated expression levels of miR‐134‐5p, miR‐107, and miR‐15a‐5p in qRT‐PCR were increased consistent with microarray analysis. These miRNAs distinguished MM and MUGS from HC significantly. Multivariate logistic analysis showed combination miR‐107, miR‐15a‐5p with Hb, the AUC was 0.954 (95% CI: 0.890‐1.000), sensitivity of 91.3%, and specificity of 93.7% for distinguishing MM from MUGS.

**Conclusions:**

These data demonstrate that miR‐134‐5p, miR‐107, and miR‐15a‐5p are potential diagnostic biomarkers in MM and MUGS. Moreover, the combination miR‐107 and miR‐15a‐5p with Hb can distinguish MM from MUGS.

## 1 INTRODUCTION

Multiple myeloma (MM) is a B‐cell malignancy characterized by clonal expansion of plasma cells in the bone marrow and present with typical clinical manifestations, including hypercalcemia, renal failure, anemia, and bone lesions.^1^ Monoclonal gammopathy of undetermined significance (MGUS), a pre‐malignant condition, which progresses to MM at rate of 1% per year.^2^ Early treatment can decrease M‐protein level in MUGS patients.^3^ Although serum M‐protein and bone marrow plasma cells are used for diagnosis and discrimination of MM and MGUS, these existing markers are limited. Moreover, bone marrow aspiration does not reflect tumor burden and is generally considered unacceptable. So the search for minimally invasive and more effective biomarkers has been paid more attention.

MicroRNAs (miRNAs) are small non‐coding single‐stranded RNAs that negatively regulate specific target gene mRNAs by binding with 3′‐end non‐translation region of mRNA. They regulate the expression of target genes at the post‐transcriptional concentration through base pairing to partially or fully complementary sites.^4^ Individual miRNA can target multiple mRNAs and participate in and regulate cell proliferation, apoptosis and immune response.^5^ Studies have shown that miRNAs regulate critical processes in tumor initiation and progress by targeting oncogenes or tumor suppressor genes.^6^ They exist in a stable form in plasma and serum, so circulating miRNA expression profiles could be served as novel biomarkers for disease diagnosis, progression, and prognosis.^7^

In the present study, we used microarray analysis to identify miRNAs that were differential expressed in MM, MUGS, and health individuals. Then, quantitative real‐time PCR (qRT‐PCR) was used to assess the abundance of miRNAs in each sample. Using these methods, we found three miRNAs as potential diagnostic biomarkers in MM and MUGS. Multivariate logistic analysis showed combination miRNAs and serum parameter can effective discriminate MM from MUGS.

## 2 MATERIALS AND METHODS

### 2.1 Patients and controls

All MM patients, MUGS patients, and healthy controls (HC) were from Beijing Shijitan Hospital of Capital Medical University. The HC group was healthy individuals. Multiple myeloma and MUGS patients were diagnosed according to the National Comprehensive Cancer Network (NCCN) and the International Myeloma Working Group (IMWG) criterion, respectively.^8^, ^9^ For miRNA Arrays, 6 MM, 6 MUGS, and 6 HC samples were used. Six samples for each group were mixed into three. As a qRT‐PCR testing, 23 MM, 16 MUGS, and 18 HC samples were used. The clinical information of MM, MUGS, and HC were shown in Table 1. After collected in coagulation tubes, venous blood was centrifuged at 3500 *g* for 10 minutes and then removed into RNase/DNase‐free tubes as 0.5 mL aliquots, stored at −80°C. This study was approved by the Ethics Committee of Beijing Shijitan Hospital of Capital Medical University.

**Table 1:** Clinical characteristics of MM, MUGS patients, and HC

Parameters	MM N = 23	MUGS N = 16	HC N = 18
Gender			
Male, number (%)	16 (26.1)	6 (37.5)	11 (61.1)
Female, number (%)	7 (30.4)	10 (62.5)	7 (38.9)
Age range	42‐86	33‐87	53‐79
Mean	66.5	61.6	65.6
Durie‐salmon stage			
I, number (%)	3 (13.0)	ND	ND
II, number (%)	5 (21.7)	ND	ND
IIIA, number (%)	7 (30.4)	ND	ND
IIIB, number (%)	8 (34.8)	ND	ND
Isotype			
IgG, number (%)	9 (39.1)	ND	ND
IgA, number (%)	7 (30.4)	ND	ND
Light chain only, number (%)	7 (30.4)	ND	ND
Non‐secretory, number (%)	0	ND	ND

Abbreviations: HC, health control; MM, multiple myeloma; MUGS, monoclonal gammopathy of undetermined significance; ND, not determined.

### 2.2 RNA extraction

Total RNA including miRNAs were isolated using the miRNeasy kit (Qiagen) according to the manufacturer's instructions for serum samples. The RNA was stored at −80°C until use.

### 2.3 MiRNA profiling

Using the Agilent Human, Release 21.0 (8*60K, Design ID: 070156) and analyze miRNA expression in MM, MUGS, and HC groups. Total RNA was quantified by the NanoDrop ND‐2000 (Thermo Scientific), and the RNA integrity was evaluated using Agilent Bioanalyzer 2100 (Agilent Technologies). The sample labeling, microarray hybridization, and washing were carried out according to the manufacturer's protocols. Feature Extraction software (version 10.7.1.1, Agilent Technologies) was used to analyze array images and obtain the raw data. GeneSpring software (version 13.1, Agilent Technologies) was used to finish the basic analysis with the raw data which normalized with the quantile algorithm. Hierarchical clustering was performed to display the distinguishable miRNAs expression pattern among samples.

### 2.4 MiRNA quantification

MicroRNAs were detected by using the CFX96 quantitative real‐time PCR (qRT‐PCR) System (Bio‐Rad). The total RNA was extracted from serum using TRIzol reagent (Invitrogen, 15596018) according to the instructions of the manufacturer. Using the poly (A) polymerase (NEB, M0276L), poly (A) tail was added to the 3′ end of RNA. Circulating DNA synthesized the first strand from 1μg of total RNA using random primers and MMLV reverse transcriptase (Vazyme, R223‐01). SnRNA U6 as internal control standard, the relative of the gene expression level was calculated by the 2^−ΔΔCt^. Each sample was measured repeatedly.

### 2.5 Statistical analysis

In order to identify the differentially expressed miRNAs, fold change as well as *P* value were calculated by Student's *t* test. The threshold set for up‐ and down‐regulated genes was fold change >2.0 and *P < *.05 considered statistically significant. The sensitivity, specificity, and area under the curve (AUC) were determined using receiver operator characteristic (ROC) analyses. Spearman bivariate *t* test was established to evaluate the correlation between miRNAs and serum parameters. A binary logistic regression model was established to calculate the multivariate diagnostic value. *P* < .05 was considered statistically significant. SPSS 22.0 was used to data statistically analyzed.

## 3 RESULTS

### 3.1 Comparison of serum parameters in MM and MUGS

Hemoglobin (Hb), thrombocytes (PLT), albumin (ALB), lactate dehydrogenase (LDH), creatinine (Cr), β_2_‐microglobulin (β_2_‐MG), C‐reactive protein (CRP), immune globulin IgG (IgG), immune globulin (IgG+ IgA+ IgM), and light chain (κ+λ) in serum (Ig/L−κ+λ) were analyzed among MM and MUGS patients (Table 2). Hemoglobin, PLT, ALB, and Cr in MM were lower than those in MUGS. However, Hb demonstrated significant differences (*P* < .05). On the contrary, LDH, CRP, IgG, and immune globulin were increased in MM patients, while β_2_‐MG and light chain (κ+λ) in serum showed significantly higher than those in MUGS (*P* < .05).

**Table 2:** Concentration of serum parameters in MM and MUGS

Parameter	MM (N = 23) median	MUGS (N = 16) median	*P* value
Hemoglobin (g/L)	92.96	125.56	**.000**
Thrombocytes (count ×10^9^/L)	158.56	191.44	.198
Albumin (g/L)	34.53	49.60	.094
Lactate dehydrogenase (U/L)	175.30	111.19	.050
Creatinine (μmol/L)	174.61	191.44	.701
β_2_‐microglobulin (mg/L)	9.33	2.64	**.047**
C‐reactive protein (mg/L)	24.16	3.16	.144
IgG (g/L)	19.26	11.28	.183
IgG+ IgA+ IgM (g/L)	21.56	11.28	.087
Ig/L−κ+λ (g/L)	7.84	3.82	**.044**

Note

The black font represents *P* < .05.

Abbreviations: MM, multiple myeloma; MUGS, monoclonal gammopathy of undetermined significance.

### 3.2 MiRNA profiling and analyzing

**Figure 1:**
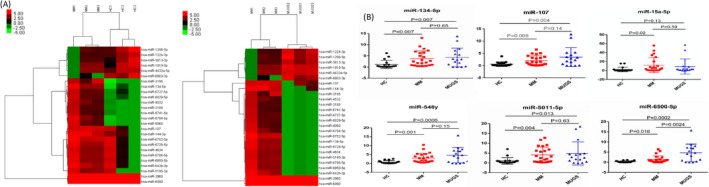
MiRNA profiling and validation. Expression patterns of 26 miRNAs in MM, MUGS, and HC (A). The miRNA expression profiles were analyzed by Agilent platform. Comparison of the serum relative expression levels of miRNAs in HC, MM, and MUGS (B). The relative expression of miRNAs were carried out by qRT‐PCR. The data were obtained from at least two independent experiments

The miRNA expression profiles were normalized based on spiked‐in controls spotted in the Agilent platform. MM, MUGS, and HC samples were identified to express miRNA patterns differentially. Eighty‐six miRNAs were significantly dysregulated (*P* < .05, fold change >2.0) between MM patients and HC. Among them, 32 miRNAs were up‐regulated, and 54 miRNAs were down‐regulated in MM. Moreover, 89 miRNAs expression in MM were distinct from MGUS. In detail, 73 miRNAs were up‐regulated, and 16 miRNAs were down‐regulated. Of note, 26 miRNAs in MM were aberrantly expressed when compared with either MUGS or HC (Figure 1A). In MM, 10 miRNAs were significantly differential expressed (*P* < .005, fold change >30.0) including five were up‐regulated (miR‐134‐5p, miR‐107, miR‐15a‐5p, miR‐5159‐3p, miR‐1914‐3p) and five down‐regulated miRNAs (miR‐4723‐3p, miR‐5588‐3p, miR‐6893‐3p, miR‐7106‐3p, miR‐6722‐5p).

Furthermore, we also compared the profile of miRNAs between MGUS and HC. Eighteen aberrant miRNAs were identified in MGUS (*P* < .05, fold change >2.0): 7 were up‐regulated and 11 were down‐regulated (data not shown).

### 3.3 Validation of miRNAs using qRT‐PCR

Out of the top 10 dysregulated miRNAs in MM, three up‐regulated miRNAs (miR‐134‐5p, miR‐107, and miR‐15a‐5p) miRNAs were further validated, based on their fold change and *P* value. In addition, three downgrading miRNA (miR‐548y, miR‐6500‐5p, and miR‐5011‐5p) we concerned were verified. The data were shown in Figure 1B. For miR‐134‐5p and miR‐107, the levels were higher in MM and MGUS compared with HC (*P* < .01). The level of miR‐15a‐5p was higher in MM compared with HC (*P* < .05). Similarly, it was higher in MUGS. The levels of miR‐134‐5p, miR‐107, and miR‐15a‐5p were no difference between MM and MUGS (*P* > .05).

Although there was a significant difference in expression of miR‐548y, miR‐6500‐5p, and miR‐5011‐5p in MM and MUGS compared with HC (*P* < .05), the elevated expression was contrary to the microarray results. Therefore, these three miRNAs were excluded from further analysis.

### 3.4 Correlate analysis of miRNAs in MM and MUGS

**Figure 2:**
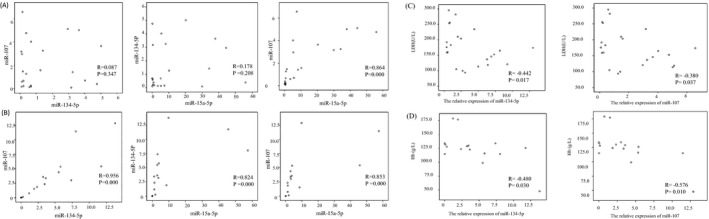
Correlation analysis of miR‐134‐5p, miR‐107, and miR‐15a‐5p was performed in MM (A) and MUGS (B). Correlation analysis of miR‐134‐5P and miR‐107 with LDH in MM (C). Correlation analysis of miR‐134‐5P and miR‐107 with Hb in MUGS (D)

Spearman bivariate *t* test was used to calculate the correlation between miR‐134‐5p, miR‐107, and miR‐15a‐5p. In MM, a significantly positive correlation was detected between miR‐107 and miR‐15a‐5p (*r* = .864, *P* = .000). On the contrary, no correlation was observed between miR‐134‐5p and miR‐107 (*r* = .087, *P* = .347) and miR‐15a‐5p (*r* = .178, *P* = .208), shown in Figure 2A. Interestingly, in MUGS, miR‐134‐5p was significantly correlated with miR‐107 (*r* = .956, *P* = .000) and miR‐15a‐5p (*r* = .824, *P* = .000), and miR‐107 was positively correlated with miR‐15a‐5p (*r* = .853, *P* = .000), shown in Figure 2B.

### 3.5 Correlate analysis of miR‐134‐5p, miR‐107, miR‐15a‐5p, and serum parameters

Correlation analysis was performed between miR‐134‐5p, miR‐107, and miR‐15a‐5p and abovementioned 10 serum parameters. In MM, miR‐134‐5p and miR‐107 were negatively correlated with LDH, respectively (*r* = −.442 *P* = .017, *r* = −.380 *P* = .037), shown in Figure 2C. In MUGS, miR‐134‐5p and miR‐107 showed negatively correlated with Hb, respectively (*r* = −.480 *P* = .030, *r* = −.576 *P* = .010), shown in Figure 2D.

### 3.6 ROC analysis of miR‐134‐5p, miR‐107, and miR‐15a‐5p

**Figure 3:**
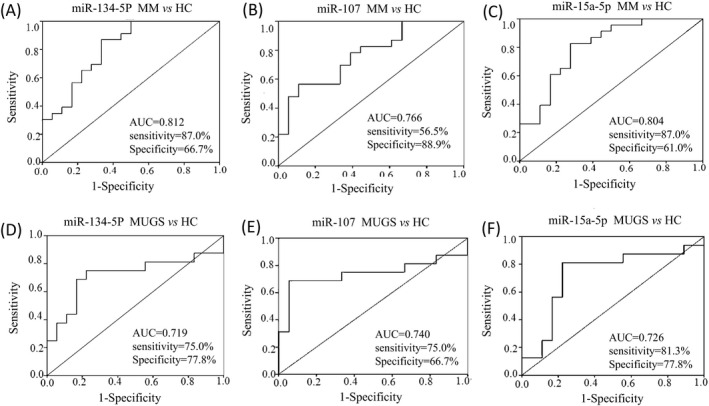
The AUC of miR‐134‐5p (A), miR‐107 (B), and miR‐15a‐5p (C) was shown respectively in MM. The AUC of miR‐134‐5p (D), miR‐107 (E), and miR‐15a‐5p (F) was shown respectively in MUGS

Receiver operator characteristic curves analysis showed that serum relative expression levels of miR‐134‐5p, miR‐107, and miR‐15a‐5p can be used to discriminate MM from HC (Figure 3A‐C). MiR‐134‐5p yielded an AUC of 0.812 (*P* = .001, 95% CI: 0.677‐0.946) with 87.0% sensitivity and 66.7% specificity at a cutoff value of 0.511. For miR‐107, with an AUC of 0.766 (*P* = .004, 95% CI: 0.621‐0.910), the sensitivity and specificity were 56.5% and 88.9%, respectively, at a cutoff value of 1.260. The AUC of miR‐15a‐5p was 0.804 (*P* = .001, 95% CI: 0.666‐0.943). At a cutoff value of 0.340, the sensitivity and specificity were 87.0% and 61.0%, individually.

Similarly, the serum relative expression of miR‐134‐5p, miR‐107, and miR‐15a‐5p can also discriminate MGUS from HC (Figure 3D‐F). MiR‐134‐5p yielded an AUC of 0.719 (*P* = .030, 95% CI: 0.529‐0.908) with 75.0% sensitivity and 77.8% specificity at a cutoff value of 1.100. For miR‐107, with an AUC of 0.740 (*P* = .017, 95% CI: 0.549‐0.931), the sensitivity and specificity were 75.0% and 66.7% correspondingly at a cutoff value of 0.612. The AUC of miR‐15a‐5p was 0.726 (*P* = .025, 95% CI: 0.541‐0.911). When at a cutoff value of 1.071, the sensitivity and specificity were 81.3% and 77.8%, respectively.

### 3.7 ROC analysis in combination with miR‐134‐5p, miR‐107, and miR‐15a‐5p

According to the correlation of miRNAs, combined miR‐107 with miR‐15a‐5p, the AUC for distinguishing MM from HC did not change significantly (AUC = 0.766, *P* = .004, 95% CI: 0.622‐0.910). Similarly, combined with miR‐134‐5p, miR‐107, and miR‐15a‐5p did not improve the discrimination between MUGS and HC (AUC = 0.712, *P* = .035, 95% CI: 0.508‐0.916). The ROC curves for each group were not shown.

### 3.8 ROC analysis of miR‐134‐5p, miR‐107, and miR‐15a‐5p to discriminate MM from MUGS

The serum relative expression of miR‐134‐5p, miR‐107, and miR‐15a‐5p cannot differentiate MM from MUGS. MiR‐134‐5p yielded an AUC was 0.489 (*P = *.909), miR‐107 yielded an AUC was 0.427 (*P* = .441), and miR‐15a‐5p yielded an AUC was 0.557 (*P* = .549). Moreover, these three miRNAs combination did not make a distinction between MM and MUGS (AUC = 0.500, *P* = .095). The ROC curves were not shown.

### 3.9 Combination miR‐134‐5p, miR‐107, and miR‐15a‐5p with serum parameters to discriminate MM from MUGS

As mentioned above, compared with MUGS, Hb in the MM was significantly decreased, while β_2_‐MG and Ig/L−κ+λ were significantly increased. In addition, miR‐134‐5p and miR‐107 in MM were negatively correlated with LDH. Multivariate logistic regression analyses, which included these three miRNAs and four abovementioned serum parameters, were used to evaluate the diagnostic value for distinguishing MM from MUGS. The result revealed that the combination miR‐107 (HR = 0.388, 95% CI: 0.202‐0.748, *P* = .005) and miR‐15a‐5p (HR = 1.124, 95% CI: 1.027‐1.231, *P* = .011) with Hb (HR = 0.867, 95% CI: 0.787‐0.955, *P* = .004) had significant value in distinguishing MM from MUGS. The diagnostic formula was:

$$Y=\text{logit(}P)=17.654-0.946X_{\text{miR}-107}+0.117X_{\text{miR}-15a-5p}-0.143X_{\text{Hb}}$$

**Figure 4:**
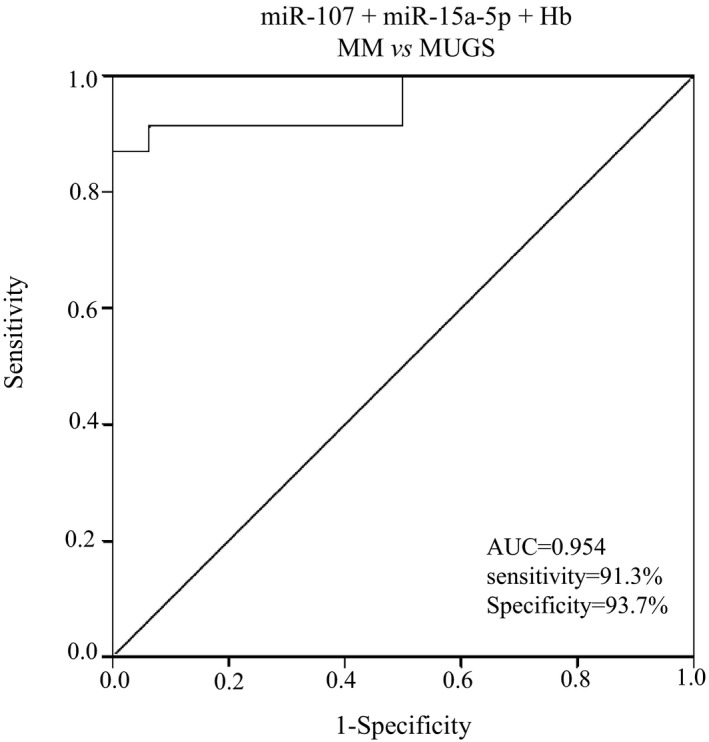
ROC analysis of miR‐107 and miR‐15a‐5p combined with Hb in the identification of MM and MUGS. The AUC for combination of miR‐107, miR‐15a‐5p, and Hb was 0.954 (95% CI: 0.890‐1.000)

The AUC of multivariate logistic regression was 0.954 (*P* = .000, 95% CI: 0.890‐1.000) with 91.3% sensitivity and 93.7% specificity at a cutoff value of 0.529. (Figure 4).

## 4 DISCUSSION

A significant number of miRNAs are present in genomic regions associated with cancer and appear as circulating molecules in human peripheral blood.^10^ Effective circulating miRNAs testing can offer potential tool for early tumor detection and allow treatment at a less advanced stage for remarkable clinical improvement.

In our study, we used the array platforms and employed normalization methods in MM and compared the miRNA relative expression levels between MUGS and HC. One hundred and seventy‐five miRNAs were abnormally expressed in MM compared with MUGS and HC. Of these, 26 miRNAs aberrantly expressed when compared with either MUGS or HC. Consequently, these dysregulated miRNAs may be potential diagnostic markers for MM.

Monoclonal gammopathy of undetermined significance is plasma cell dyscrasia, with the tendency to progress to MM. The risk factors for MGUS remain unclear. Several studies investigate molecular techniques in MUGS progression to MM.^11^, ^12^ We compared the profile of miRNAs between MGUS patients with HC: 7 were up‐regulated whereas 11 were down‐regulated. Among of 11 down‐regulated miRNAs, miR‐5011‐5p, miR‐6499‐3p, miR‐6722‐5p, and miR‐6849‐3p were also shown down‐regulated in MM (data not shown). The pathway enrichment analysis showed the target genes of these miRNAs engaged in cancer signaling pathways. Therefore, these miRNAs may play important roles in carcinogenesis at early stage and serve as indicator for MM.

In the following experiment, miR‐134‐5p, miR‐107, and miR‐15a‐5p in MM and MUGS were higher than those in HC detected by qRT‐PCR, consistent with the microarray analysis. The relative expression level of miR‐107 was correlated with miR‐15a‐5p (*r* = .864, *P* = .000) in MM significantly. Moreover, these three miRNAs were positive correlation in MUGS.

In ROC curves analysis, all of these three miRNAs showed clearly specificity and sensitivity in discriminating MM and MUGS from HC. MiR‐134‐5p and miR‐15a‐5p showed better sensitivity (87.0%, 87.0%, respectively) and miR‐107 have greater specificity (88.9%) in discriminating MM from HC. Compared with HC, the sensitivity and specificity of miR‐15a‐5p in MUGS were better than miR‐134‐5p and miR‐107, which were 81.3% and 77.8%, respectively.

MiR‐134‐5p involved in the pathogenesis and progression of hematologic malignancy has already been reported.^13^ This is the first time that it serves as a diagnostic biomarker for MM. The predicted target genes of miR‐134‐5p include ITGB1 and PIK3R1, which are involved in insulin‐like growth factor receptor (IGF‐1) signaling pathway, cell proliferation, and apoptotic process, and are related to the occurrence and development of MM.^14^, ^15^

Our results revealed that miR‐15a‐5p, belonging to 15a/16 cluster located at chromosome 13q14, was up‐regulated in MM.^16^ Several studies have reported that cytokines such as interleukin‐6 (IL‐6), IGF‐1, and vascular endothelial growth factor (VEGF) mediate the growth of MM cells and resist to apoptosis through the mitogen‐activated protein kinase (MAPK) signaling pathways and phosphatidylinositol 3 kinase/AKt kinase (PI3K‐Akt) pathways.^17^, ^18^, ^19^ Other independent research hypothesizes that overexpression of miR‐15a inactivates p53 in MM, associated with high‐risk cancer gene sets, high proliferation index, and shorter progression‐free survival (PFS).^20^, ^21^, ^22^ In contrast, other investigations demonstrate that miR‐15a participates in tumor suppression through multiple regulatory mechanisms.^23^, ^24^, ^25^ Take together, miR‐15a plays different roles in primary and advanced MM, and may engage in many roles as the disease development, which warrants further study.

Similar to miR‐134‐5p and miR‐15a‐5p, the target genes of miR‐107 has been predicted. Its high expression is positively associated with risk score and proliferation index, supporting poor clinical outcome.^26^

Our results show that miR‐134‐5p, miR‐107, and miR‐15a‐5p detected in MUGS are similar to MM and can significantly discriminate from HC. Remarkably, 3 patients in MUGS group advanced to MM within 1 year from diagnosis of MUGS. This consistently that MGUS is a pre‐cancerous state for MM. However, only these three miRNAs could not effectively identify MM and MUGS.

Serum parameters showed significant differences between MM and MUGS. Compared with MUGS, Hb was significant decreased (*P* < .05), while β_2_‐MG and light chain (κ+λ) significant increased (*P* < .05) in MM. In addition, miRNAs were negatively correlated with LDH (*P* < .05) in MM. These serum parameters reflecting the tumor load of MM, not only relate to the clinical stage, but also affect the renal function and prognosis of MM patients.^27^, ^28^, ^29^ In our previous study, we demonstrated that these serum indicators were related to MM prognosis.^30^ In present study, multivariate logistic regression analysis showed miR‐107, miR‐15a‐5p, and Hb were potential diagnostic biomarkers for the identification of MM and MUGS. With the AUC increased to 0.954, the sensitivity and specificity were 91.3% and 93.7%, respectively. The diagnostic value of MM is significantly improved.

In summary, our study indicates that miR‐134‐5p, miR‐107, and miR‐15a‐5p are up‐regulated in MM and MUGS, which could be served as potential diagnostic markers. Moreover, combination of miR‐107 and miR‐15a‐5p with Hb will distinguish MM from MUGS so as to provide early treatment and improve the prognosis.
